# Host cell wall composition and localized microenvironment implicated in resistance to basal stem degradation by lettuce drop (*Sclerotinia minor*)

**DOI:** 10.1186/s12870-024-05399-5

**Published:** 2024-07-29

**Authors:** Ivan Simko, Bullo Erena Mamo, Clifton E. Foster, Neil D. Adhikari, Krishna V. Subbarao

**Affiliations:** 1 United States Department of Agriculture, Agricultural Research Service, Sam Farr United States Crop Improvement and Protection Research Center, Salinas, CA 93905 USA; 2 Department of Plant Pathology, University of California, Davis, c/o Sam Farr United States Crop Improvement and Protection Research Center, Salinas, CA 93905 USA; 3 Great Lakes Bioenergy Research Center, Michigan State University, East Lansing, MI 48824 USA; 4 Present address: Department of Microbiology and Plant Pathology, University of California, Riverside, CA 92521 USA; 5 Present address: Pace Analytical, Wixom, MI 48393 USA; 6 Present address: California Department of Public Health, Sacramento, CA 95814 USA

**Keywords:** Lignin, Hemicellulose, Monosaccharides, Stem strength, Guaiacyl, Syringyl, Xylose

## Abstract

**Background:**

*Sclerotinia* spp. are generalist fungal pathogens, infecting over 700 plant hosts worldwide, including major crops. While host resistance is the most sustainable and cost-effective method for disease management, complete resistance to *Sclerotinia* diseases is rare. We recently identified soft basal stem as a potential susceptibility factor to *Sclerotinia minor* infection in lettuce (*Lactuca sativa*) under greenhouse conditions.

**Results:**

Analysis of stem and root cell wall composition in five *L. sativa* and one *L. serriola* accessions with varying growth habits and *S. minor* resistance levels revealed strong association between hemicellulose constituents, lignin polymers, disease phenotypes, and basal stem mechanical strength. Accessions resistant to basal stem degradation consistently exhibited higher levels of syringyl, guaiacyl, and xylose, but lower levels of fucose in stems. These findings suggest that stem cell wall polymers recalcitrant to breakdown by lignocellulolytic enzymes may contribute to stem strength-mediated resistance against *S. minor*.

**Conclusions:**

The lignin content, particularly guaiacyl and syringyl, along with xylose could potentially serve as biomarkers for identifying more resistant lettuce accessions and breeding lines. Basal stem degradation by *S. minor* was influenced by localized microenvironment conditions around the stem base of the plants.

**Supplementary Information:**

The online version contains supplementary material available at 10.1186/s12870-024-05399-5.

## Introduction

Lettuce (*Lactuca sativa*) is the world’s leading leafy vegetable but is vulnerable to diseases such as downy mildew, Fusarium and Verticillium wilts, and lettuce drop [1] caused by *Sclerotinia minor* and *S. sclerotiorum*. *Sclerotinia* spp. infect over 700 hosts worldwide [2, 3] and pose serious threats to production of lettuce and other crops [4]. Once infected, lettuce plants collapse within a few days [5, 6]. Despite efforts to control it using cultural, antifungal, and biological methods [7], lettuce drop remains challenging due to the pathogens’ ability to overwinter in soil and windborne nature [8, 9]. Host resistance is the most effective strategy of managing *Sclerotinia* diseases, but it is limited in economically important crops [10]. Although, a complete resistance of lettuce to *S. minor* has not been identified, some cultivars exhibit partial resistance linked to certain developmental traits [11]. These traits, including plant foliar density, rosette architecture, small leaf area, altered growth habit, plant height, rapid bolting, flowering and maturity [11–15], are often considered undesirable in modern lettuce cultivars [16–19].

*Sclerotinia* spp. have evolved mechanisms to overcome host immunity and procure nutritional sources [20, 21], including the production of phytotoxins and host cell wall-degrading enzymes (CWDEs). These enzymes facilitate rapid infection and nutrient acquisition [20, 22] by degrading host tissues [23], including the stem. In other crop species, stem mechanical strength is strongly correlated with resistance to lodging and soil-borne fungal diseases [24–27]. Soft basal stem have also been recently identified as a plausible susceptibility factor to *S. minor* in lettuce, leading to what we term ‘plant architecture- or stem strength-mediated resistance’ (PAMR) [14]. However, the causal relationship between stem mechanical strength and resistance to lettuce drop remain largely elusive. Given the absence of basal stem and root degradation in lettuce accessions with strong stems [14], we hypothesize that this may be due to differences in host tissue composition or cell wall architecture [28].

The plant cell wall acts as the first line of defense against pathogens [29–32]. Cellulose, hemicellulose, and lignin, the primary components of the cell wall, contribute to its structural integrity [24, 33, 34], playing an essential role in disease resistance [24, 30, 35, 36]. Several studies have demonstrated a positive correlation between lignin content and resistance to *Sclerotinia* spp. in *Brassicaceae* (*Brassica napus*, *B. oleracea*, and *Camelina sativa*) [37–43]. For instance, significant differences in the constitutive expression and regulation patterns of genes involved in the synthesis of lignin monomers (syringyl and guaiacyl) were observed in response to *S. sclerotiorum* infection in false flax (*C. sativa*) [40]. In oilseed rape (*B. napus*), the transcript abundance of a gene catalyzing a step in the lignin biosynthesis pathway decreased in a susceptible cultivar inoculated with *S. sclerotiorum* [41]. Furthermore, in oilseed rape cultivars transformed with cDNA expressing recombinant proteins involved in lignin biosynthesis, there was an increase in the lignin content which correlated with enhanced resistance to *Sclerotinia* [39]. In cabbage (*B. oleracea*), cell wall degradation was inhibited in F_2_ population resistant to *S. sclerotiorum* [43].

However, contradictory results have also been reported regarding the role of lignin in host resistance to *Sclerotinia* spp. Some studies show a negative correlation or unreliable prediction of resistance based on stem lignin content, as observed in soybean (*Glycine max*) [44], peanuts (*Arachis hypogaea*) [45], and oilseed rape [46]. In soybean infected with *S. sclerotiorum*, resistance appears to be regulated by reprograming of the phenylpropanoid pathway, diverting its flux from lignin to lignin-intermediates, anthocyanins and phytoalexins [47]. This suggests that lignin intermediates, such as caffeic and ferulic acid, along with other compounds with antifungal activity (e.g., phytoalexins﻿), are crucial components of the resistance response. The results were also confirmed at the transcript level, where several genes, including those encoding phenylalanine ammonia-lyase (PAL), lignin biosynthetic enzymes, chalcone synthase (CHS), and flavonol synthase (FLS), were down-regulated in the resistant line [47].

To investigate the biological basis of PAMR to *S. minor* in lettuce, we analyzed the carbohydrate polymers, monosaccharides, lignin, and monolignol composition in plant’s basal stems and roots. Additionally, we compared the cell wall composition of lettuce drop-tolerant and susceptible accessions to understand the progression of basal stem degradation after *S. minor* infection. Furthermore, we quantified fungal DNA in the basal stems of lettuce accessions to determine the influence of canopy architecture on pathogen growth and development.

## Materials and methods

### Plant material

Six accessions representing different horticultural types of lettuce were used for the study: Eruption (Latin type), Reine des Glaces (Batavia subtype of crisphead lettuce), Salinas (iceberg subtype of crisphead lettuce), Da Ye Wo Sun (stem type), PI 251246 (primitive oil-seed type), and 11-G99 (*L*. *serriola*, the wild progenitor of cultivated lettuce). These accessions exhibit varying rates of stem elongation and field reactions to lettuce drop. Eruption, Reine des Glaces, Salinas, and 11-G99 (in fall) are slow-bolting. Da Ye Wo Sun and 11-G99 (in spring/summer) have intermediate rates of stem elongation; while PI 251246 bolts rapidly. Eruption, PI 251246, and 11-G99 (in spring/summer) are partially resistant to lettuce drop, although Eruption shows high disease severity once infected. Reine des Glaces, Salinas, Da Ye Wo Sun, and 11-G99 (in fall) are susceptible to lettuce drop. Seeds of all accessions were obtained from the USDA-ARS lettuce germplasm collection in Salinas, California, USA.

### Inoculation and sampling

Plants were grown in a greenhouse in sterilized soil mixture (2:1 ratio of sand to potting mix) at 20–25 °C during the day and 15–17 °C at night. Inoculation with *S. minor* mycelia followed a previously published protocol [14]. Two independent experiments were performed using a completely randomized design, with two replications and one plant per accession and replicate. For inoculation, ten *S. minor*-infested rye seeds were placed a few millimeters under the soil surface next to the plant’s basal stem. To ensure that the accessions were compared at the same developmental stage, inoculation took place when plants reached approximately 50% flowering. For analyses of cell wall composition, plants were sampled at three stages: at the time of inoculation (control, healthy plants), six to seven days post-inoculation (early phase of disease development), and 14 to 20 days post-inoculation (late sampling at an advanced phase of disease development). This sampling approach was designed to mimic the two-phase infection model proposed for a closely related *S. sclerotiorum*, in which the pathogen suppresses defense reactions in the early phase and induces host necrosis and cell wall degradation in the late phase [23].

Stem sampling involved cutting each plant at the stem base, and 8.5 cm of the stem above the base was harvested. The sampling of 8.5 cm of stem tissue (from the base to 8.5 cm above) was chosen because this area represents the stem region in contact with the pathogen and thus is the primary site of infection. This sampling length is based on our experience with the pathogen infecting lettuce in both field and greenhouse conditions. For sampling underground parts, the entire root system was removed from the pot, rinsed under running water to remove any cultivation media, and then placed into 50 mL tubes. Samples were frozen in liquid nitrogen, lyophilized, and stored at -20 °C until processing.

### Cell wall composition analysis

Lyophilized stem and root samples were ground using a Thomas Wiley Mini-Mill, 40 mesh (Thomas Scientific, Swedesboro, New Jersey, USA). A 100–200 mg of ground sample was submitted for analysis. Sixty mg aliquots of the ground, dried biomass were ball milled with the iWall grinding and feeding robot [48] for primary (cellulose and hemicellulose) and secondary (lignin) cell wall composition analyses [49, 50]. The milled material was used to prepare the alcohol insoluble residue (AIR) by sequential extraction with water, 70% ethanol, and 1:1 chloroform: methanol to remove the soluble components, such as sugars, proteins, lipids, pigments, DNA, and RNA. The AIR was then treated with amylase and pullulanase in a 37 °C rotisserie incubator with end-over-end rotation for approximately 12 h and washed with water to remove the starch content, yielding isolated lignocellulosic material for cell wall analysis.

The isolated lignocellulosic cell wall material was dried and weighed into three 2 mg technical replicates for matrix polysaccharide (hemicellulose) composition and crystalline cellulose assays. Polysaccharide composition was analyzed via GC-MS (Agilent 7890A GC / 5975C MS, Agilent Technologies, Santa Clara, California, USA) after 2 M trifluoroacetic acid (TFA) hydrolysis and subsequent alditol acetate derivatization of neutral monosaccharides present in the hydrolysate. Crystalline cellulose was isolated and purified from the insoluble residue remaining from the TFA hydrolysis and then hydrolyzed in 72% sulfuric acid. The crystalline cellulose content of the lignocellulosic material was determined using the colorimetric anthrone assay.

For lignin content analysis, the cell wall material was weighed into three 2 mg technical replicates and assayed using the acetyl bromide soluble lignin (ABSL) method [49]. The ABSL method involved treating cell wall material with a 25% (v/v) solution of acetyl bromide in glacial acetic acid at 50 °C for 3 h to solubilize the lignin matrix. The solubilized lignin was diluted with glacial acetic acid and assayed using a photospectrometer at 280 nm (Spectramax 384 plus, Molecular Devices, San Jose, California, USA). The lignin content was calculated using a molar extinction coefficient of 18.21 g^− 1^Lcm^− 1^ [49].

Lignin monomers composition analysis was performed using the thioacidolysis method, which detects the *p*-hydroxylphenyl, guaiacyl, and syringyl monomers incorporated in the lignin matrix via β-O-4 ether linkages. Dried and isolated lignocellulosic material was weighted onto three 2 mg replicates, to which a mixture of 87.5% (v/v) dioxane, 10% (v/v) ethanethiol, and 2.5% (v/v) boron trifluoride diethyl etherate was added and heated at 100 °C for 4 h to liberate the lignin monomers. The extracted thioether derivatized monomers were derivatized with N, O-bis[trimethylsilyl]acetamide (BSA) and quantitated using GC-MS analysis (Agilent 7890A GC / 5975C MS, Agilent Technologies, Santa Clara, California, USA) according to the published procedures [51].

The composition analyses provided information about the concentrations of crystalline cellulose (term cellulose used henceforth), seven neutral monosaccharides (arabinose, fucose, galactose, glucose, mannose, rhamnose, and xylose), soluble lignin (acetyl bromide soluble lignin, ABSL) and lignin monomers composition (*p*-hydroxyphenyl – term hydroxyphenyl used henceforth, guaiacyl, and syringyl). Yields of all compounds were reported in µg per mg cell wall unit. Samples of *S. minor* mycelium and sclerotia, and wood chip were included as controls in the composition analysis; samples of *Populus trichocarpa* (black cottonwood) were included in the ABSL analysis only. The *S. minor* sclerotia submitted for cell wall composition analysis were produced as previously described [13] but on rye seeds.

### Fungal DNA quantification to study effect of stem base microenvironment on *Sclerotinia* biomass

Twenty-five to thirty plants of two susceptible accessions (Da Ye Wo Sun and Reine des Glaces) and two resistant accessions (11G-99 and PI 251246) were grown in the greenhouse as described above. Plants were prepared for inoculation at approximately 50% flowering, following a completely randomized design with three replications (biological samples) for each treatment. After inoculation with rye seeds infested with *S. minor* mycelia, basal stems were either left uncovered (‘uncovered’ group), wrapped with the plant’s own leaves (‘leaves’ group), or wrapped with clear plastic wrap (Saran Wrap, SC Johnson, Mt. Pleasant, Wisconsin, USA) (‘plastic’ group). Three groups of uninoculated plants were also grown from each accession to serve as controls for the ‘uncovered’, ‘leaves’, and ‘plastic’ groups, respectively. The experiment was conducted twice following the inoculation procedure outlined previously [14]. Five to six days post-inoculation, 8.5 cm of the basal stem tissues from inoculated and uninoculated plants were harvested, frozen in liquid nitrogen, lyophilized, and stored at -20°C until processing.

Sample preparation for quantification of fungal DNA [52] were performed using a standard SDS-based genomic DNA extraction protocol. Lyophilized tissue was ground to a fine powder and genomic DNA was extracted using an extraction buffer containing Tris, NaCl, EDTA, and SDS [53]. The extracted DNA was precipitated with isopropanol, washed with ethanol, and resuspended in TE buffer. The DNA was then cleaned using the Zymo Genomic DNA Clean & Concentrator-25 kit (Zymo Research, Orange, California, USA), and quantitated using the NanoDrop 8000 spectrophotometer (Thermo Fisher Scientific, Wilmington, Delaware, USA). *S. minor* gDNA from mycelial culture used for plant inoculation served as a positive control.

Duplicate 20-µl qPCR reactions were performed with 10 ng gDNA, 2× TaqMan Fast Universal PCR master mix (Applied Biosystems, Waltham, Massachusetts, USA), 18 µM primers (SMLcc2-1 F: 5’-CGGTTGAGAACTCCACTATAACC-3’ and SMLcc2-1 R: 5’-AAGCTTCCCTTCTGACGAATAC-3’), and 5 µM of probe (SMLcc2-1 5’-(FAM)-TCCGATAGCGCACCGAATCTCAAA-(TAMRA)-p-3’). The 285 bp amplicon targeted *S. minor* laccase CDS. Reactions were run on a Roche LightCycler 480 (Roche Diagnostics, Indianapolis, Indiana, USA) with 95 °C for 10 min, 45 cycles of 95 °C for 10 s, 60 °C for 30 s. Standard curves were generated using 0.001 to 100 ng *S. minor* DNA with a 10-fold increase (Additional file S1). Specificity was tested against non-target pathogens (*Verticillium dahliae* isolate VdLs17, *Botrytis cinerea*, and *Aspergillus niger*) and lettuce accessions. Water controls were used to confirm no contamination. The pathogen DNA was quantified from mean Ct values of duplicates, subtracting traces of fungal biomass detected in uninoculated controls.

### Statistical analyses

Statistical analyses were conducted using JMP Pro 17 software (SAS Institute, Cary, North Carolina, USA). The methods included analysis of variance (ANOVA) with *post-hoc* Tukey’s honestly significant difference (Tukey’s HSD) test, *t*-test, Pearson linear correlation, and principal component analysis (PCA). Differences in compound content were analyzed using a mixed-effect ANOVA model, with resistant and susceptible groups as fixed effects and accessions as random effects. The number of days to reach five disease progress stages (mycelium emergence, lower leaf discoloration, leaf wilting, shoot wilting, and plant mortality), previously published [14], was converted into the area under the disease progress stairs (AUDPS) [54]. AUDPS values were then correlated with stem and root cell wall composition data collected at the control (healthy plants), early, and late phases of disease development. *P*-values for correlations were adjusted for multiple comparisons using false discovery rate (FDR) method implemented in JMP Pro 17 software. PCA was performed using resistance or composition data from all evaluated plants of six accessions.

To assess the effects of microenvironmental conditions and nutrient availability on fungal DNA quantity (pathogen growth/development), partial eta squared (*η*^2^) values were calculated from ANOVA to estimate effect sizes. A simplified model included three main factors: resistance phenotype (susceptible or resistant), microenvironmental conditions (wrapped or uncovered), and nutrient availability (leaves or no leaves). The ‘plastic’ and ‘leaves’ wrapping treatments were assumed to provide similar microenvironmental conditions but differed in nutrient availability due to the presence of leaf tissue. The ’uncovered’ treatment was assumed to have similar nutrient availability to the ‘plastic’ treatment but with different microenvironmental conditions. However, this simplified model was incomplete as no treatment provided the same microenvironmental conditions as the ‘uncovered’ treatment while having nutrient availability similar to the ‘leaves’ treatment.

## Results

Significant differences among lettuce accessions were observed in disease progression, stem and root degradation after inoculation with *S. minor* in a greenhouse [14]. Stem mechanical strength was correlated with the outcome of *S. minor* infections [14] (Fig. S1), prompting detailed analyses of stem and root cell wall composition to identify compounds related to delayed disease symptoms, reduced degradation, and higher stem strength observed in 11-G99 and PI 251246 (Fig. S2).

Analysis of monosaccharides determined that stem cell walls were primarily composed of xylose, followed by glucose, arabinose, galactose, rhamnose, mannose, and fucose (Table 1). Root cell walls exhibited a similar composition with xylose being the most abundant (Table 1). Among the three monolignols quantified in stems, syringyl had the highest content, followed by guaiacyl, and hydroxyphenyl. Guaiacyl was the most abundant in root cell walls, followed by syringyl, and hydroxyphenyl (Table 1).

**Table 1:** Cell wall composition of lettuce stems and roots, *S. minor* mycelium and sclerotia, and wood chips

Compound^a^	Lettuce stem	Lettuce root	*S. minor* mycelium	*S. minor* sclerotia^b^	Wood chips^c^
Rhamnose	9.70 ± 0.72	4.77 ± 0.52	0.38	0.93	1.79
Fucose	1.28 ± 0.09	0.80 ± 0.08	0.01	1.00	0.54
Arabinose	17.72 ± 1.93	9.21 ± 1.04	2.02	0.76	9.39
Xylose	128.08 ± 8.37	48.38 ± 7.11	2.12	1.28	21.46
Mannose	8.20 ± 0.38	5.77 ± 0.53	70.55	48.79	13.16
Galactose	14.82 ± 1.02	8.77 ± 0.91	18.23	45.79	5.77
Glucose	30.08 ± 2.37	18.93 ± 2.85	258.01	264.23	10.17
Cellulose	363.09 ± 9.88	146.39 ± 15.19	0.00	305.90	163.23
ABSL	127.94 ± 5.71	94.24 ± 7.48	56.51	12.74	135.13
Syringyl	30.33 ± 2.93	7.69 ± 1.51	0.14	0.33	0.86
Guaiacyl	15.77 ± 1.11	9.22 ± 1.14	0.19	0.17	10.28
Hydroxyphenyl	0.09 ± 0.01	0.10 ± 0.01	0.00	0.00	0.73

^a^ Content of all compounds is expressed in µg per mg. Lettuce composition data are pooled from across four susceptible and two resistant accessions. Data for individual accessions and three disease progression stages can be found at Table S2

^b^*S. minor* sclerotia were produced on rye seeds; therefore, the composition may be affected by debris coming from the seeds. The cellulose assay was validated for plant cell wall analysis solely; fungal composition is provided as a reference

^c^ Wood chips were from the potting mix used for growing plants and originated from an undetermined tree species. In addition, black cottonwood (*Populus trichocarpa*) chips were also added as control to analyses of acetyl bromide soluble lignin (ABSL). Two tested samples contained 24.48 and 24.49 µg/mg ABSL.

Three-way ANOVA revealed significant differences (*p* ≤ 0.05) among accessions in the content of all compounds except for mannose and glucose. Differences between stem and root composition were significant for all compounds except for hydroxyphenyl, and significant changes occurred in the content of all compounds as the disease progressed (Table S1, S2).

**Fig. 1:**
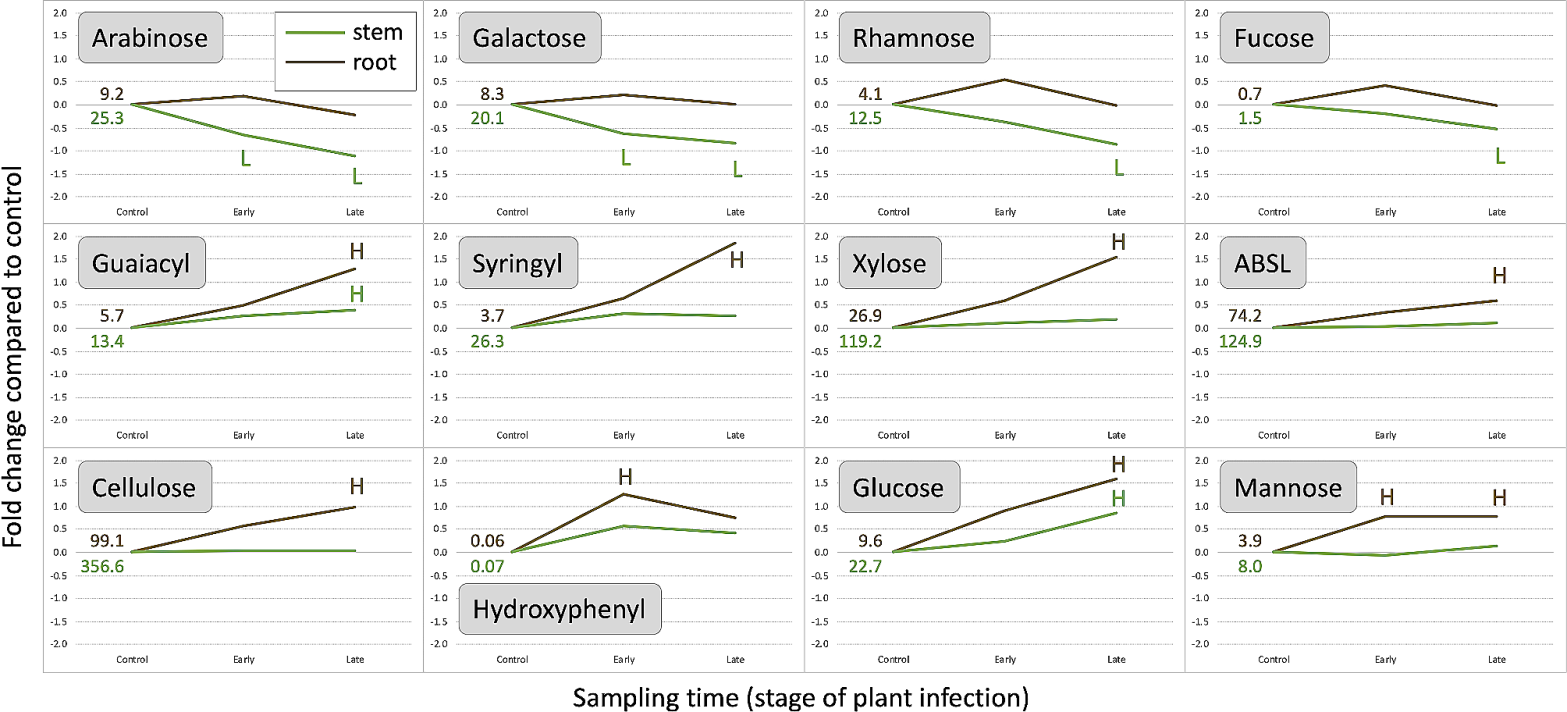
Relative changes in cell wall composition across all accessions during disease progression. Stem and root samples were collected before inoculation (control) and at early and late disease stages after inoculation with *S. minor*. Logarithmic scales show changes compared to the control. Numbers represent the average content of compounds (µg per mg) in stems (green) and roots (brown) of healthy plants. ‘H’ and ‘L’ indicate values significantly (*p* ≤ 0.05) higher or lower than the control, respectively

In stems, disease progressions led to a significant decrease in arabinose, fucose, galactose, and rhamnose, and a significant increase in glucose and guaiacyl (Fig. 1). In roots, disease progression was accompanied by a significant increase in glucose, mannose, xylose, cellulose, ABSL, hydroxyphenyl, guaiacyl, and syringyl (Fig. 1). Correlation analyses performed on both tissue types from all accessions sampled at the three disease progression stages confirmed these trends (Fig. S3).

**Fig. 2:**
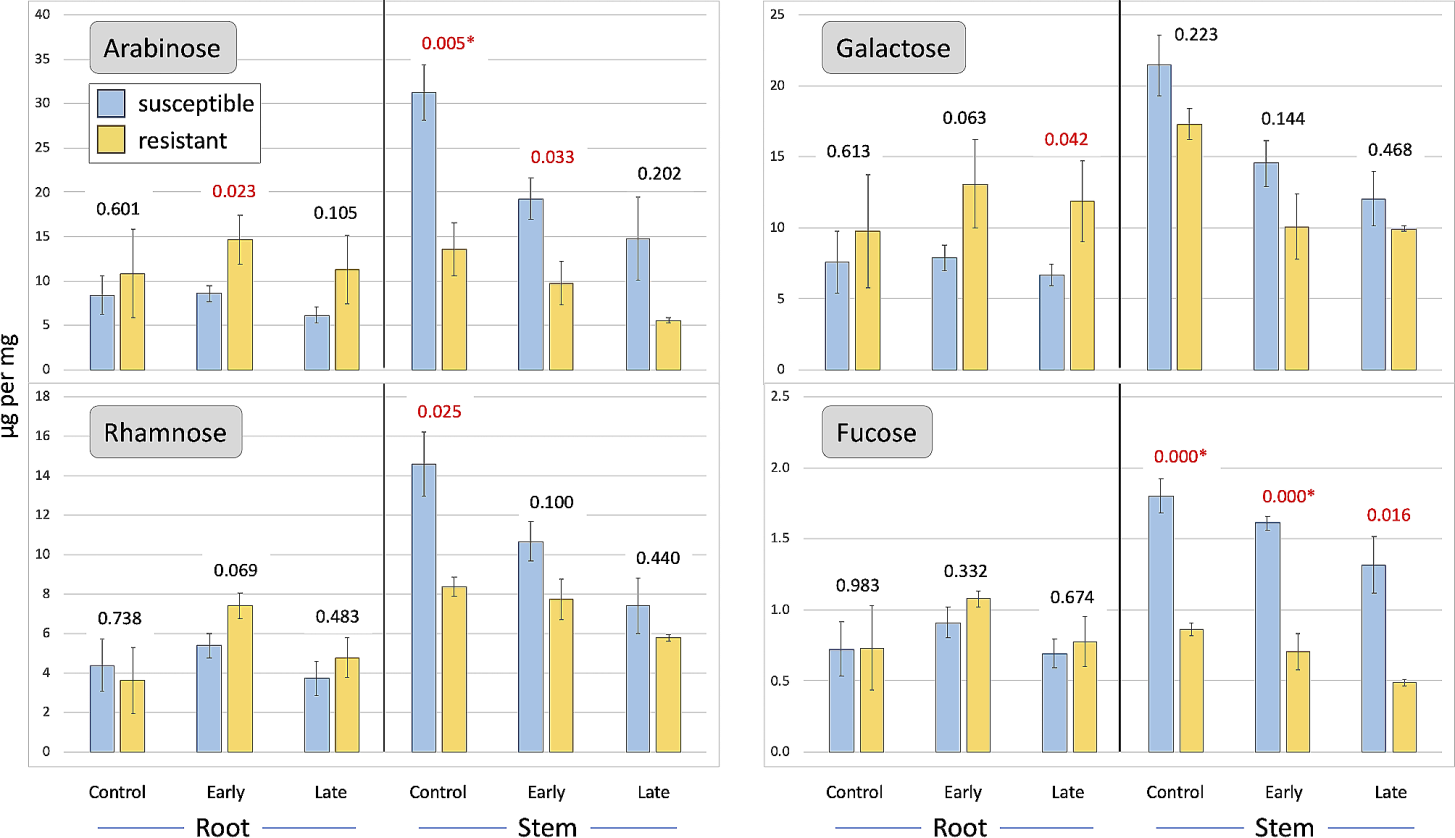
Changes in arabinose, galactose, rhamnose, and fucose content in cell walls. Samples from susceptible and partially resistant groups were collected at three disease progression stages (control, early, late) after inoculation with *S. minor*. Numbers above bars are *p*-values calculated between two phenotypic groups differing in resistance (*p* < 0.0004 is shown as 0.000). Values in red are significant at *p* ≤ 0.05, asterisks indicate values significant after false discovery rate adjustment

**Fig. 3:**
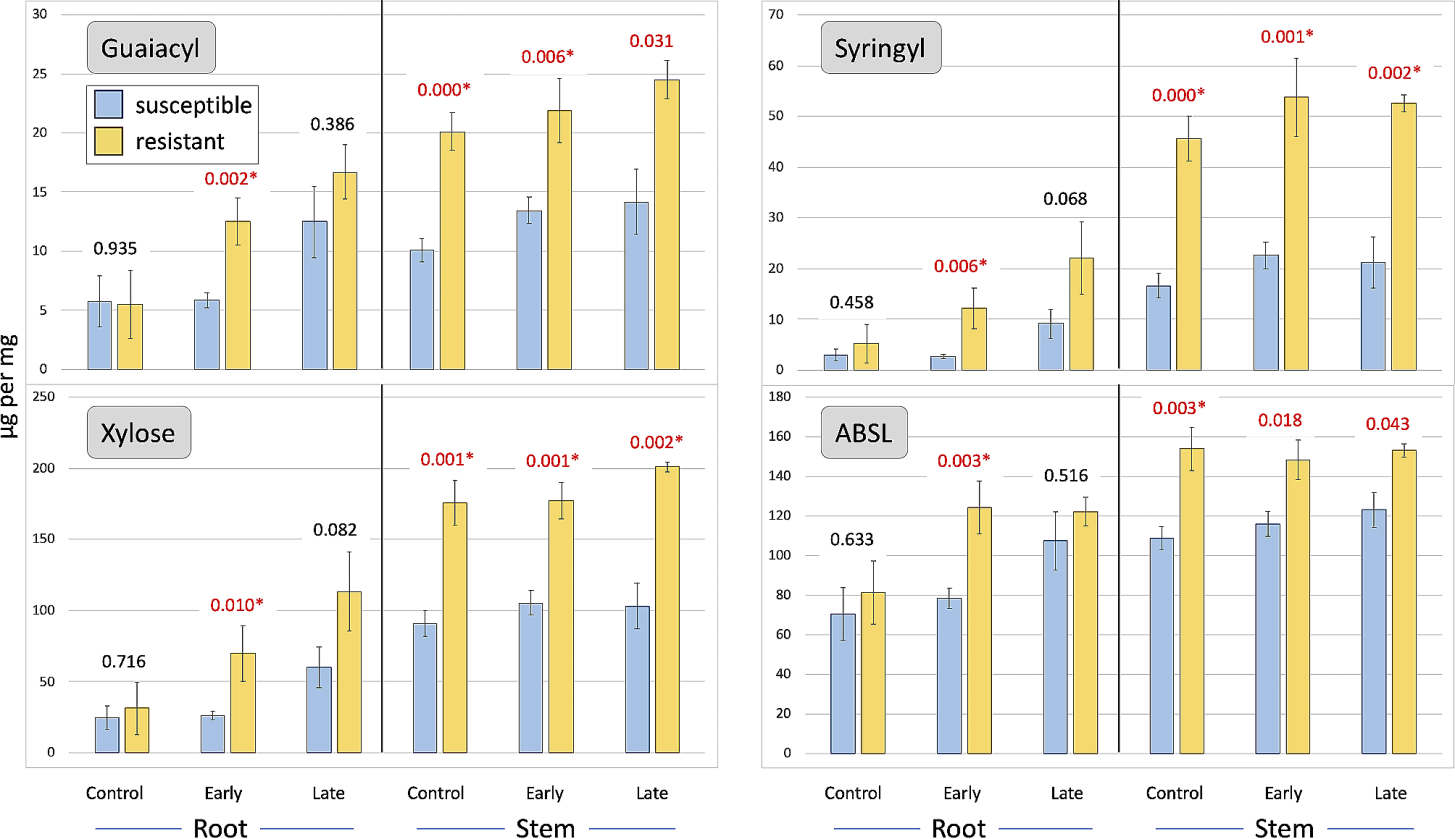
Changes in guaiacyl, syringyl, xylose, and ABSL content in cell walls. Samples from susceptible and partially resistant groups were collected at three disease progression stages (control, early, late) after inoculation with *S. minor*. Numbers above bars are *p*-values calculated between two phenotypic groups differing in resistance (*p* < 0.0004 is shown as 0.000). Values in red are significant at *p* ≤ 0.05, asterisks indicate values significant after false discovery rate adjustment

**Fig. 4:**
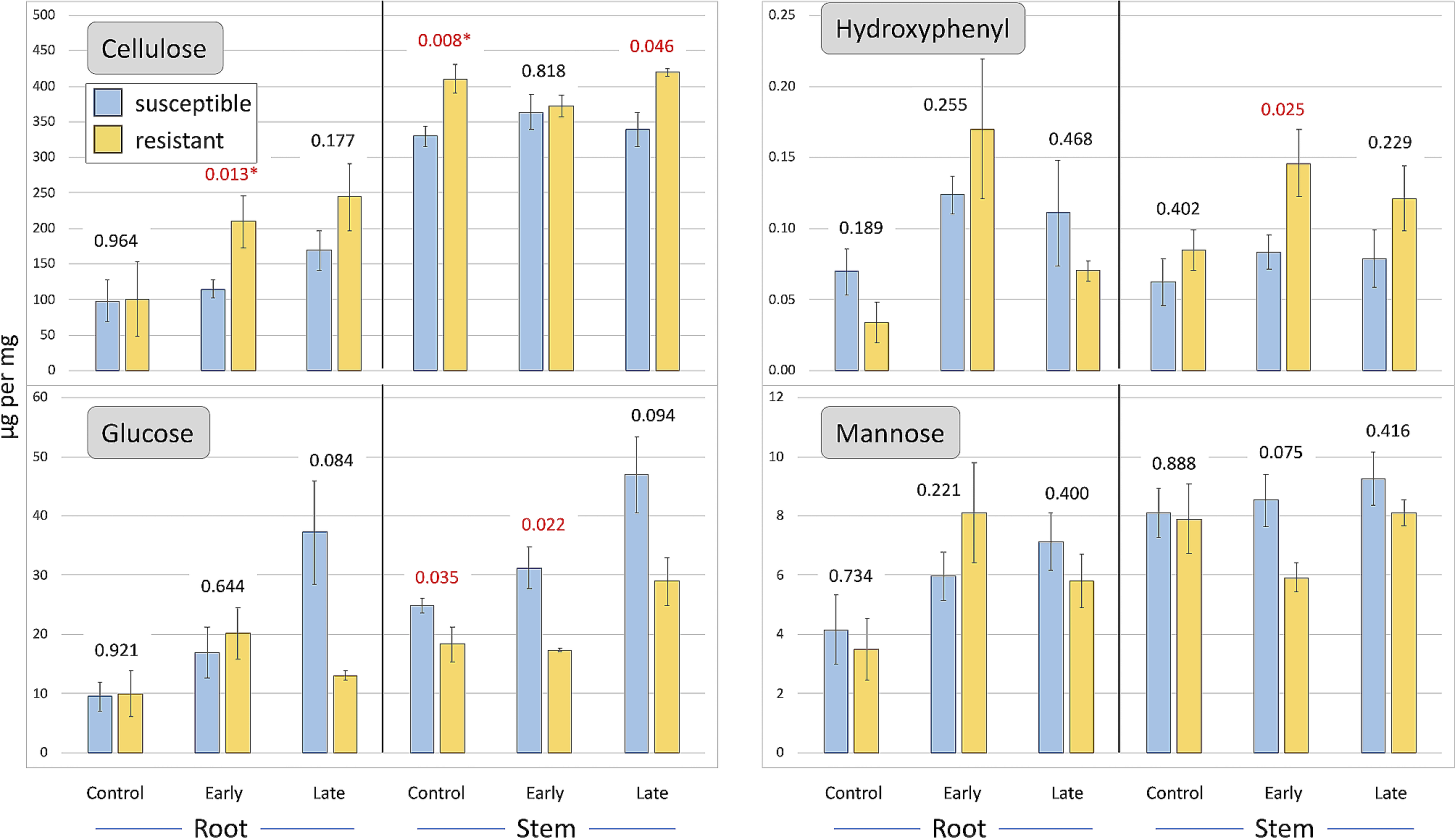
Changes in cellulose, hydroxyphenyl, glucose, and mannose content in cell walls. Samples from susceptible and partially resistant groups were collected at three disease progression stages (control, early, late) after inoculation with *S. minor*. Numbers above bars are *p*-values calculated between two phenotypic groups differing in resistance (*p* < 0.0004 is shown as 0.000). Values in red are significant at *p* ≤ 0.05, asterisks indicate values significant after false discovery rate adjustment

Comparing resistant (11-G99 and PI 251246) and susceptible (Da Ye Wo Sun, Eruption, Reine des Glaces, and Salinas) accessions, significant differences in cell wall composition were observed in stems and roots and at all disease progression stages (Figs. 2, 3 and 4). Before inoculation, heathy stems of resistant accessions had higher levels of syringyl (274%), guaiacyl (199%), xylose (193%), ABSL (142%), and cellulose (125%), but lower levels of arabinose (43%) and fucose (48%) (Figs. 2, 3 and 4). In the early stage of disease development, significant differences were still observed for syringyl, xylose, guaiacyl, and fucose, while at a later stage for syringyl and xylose (Fig. 3). In roots, differences in cell wall composition were observed only in the early stage of disease development for syringyl (462%), xylose (265%), guaiacyl (214%), cellulose (183%), and ABSL (159%) (Figs. 3 and 4). Similar trends were observed in the late stage of disease progression, but the differences were not significant.

**Fig. 5:**
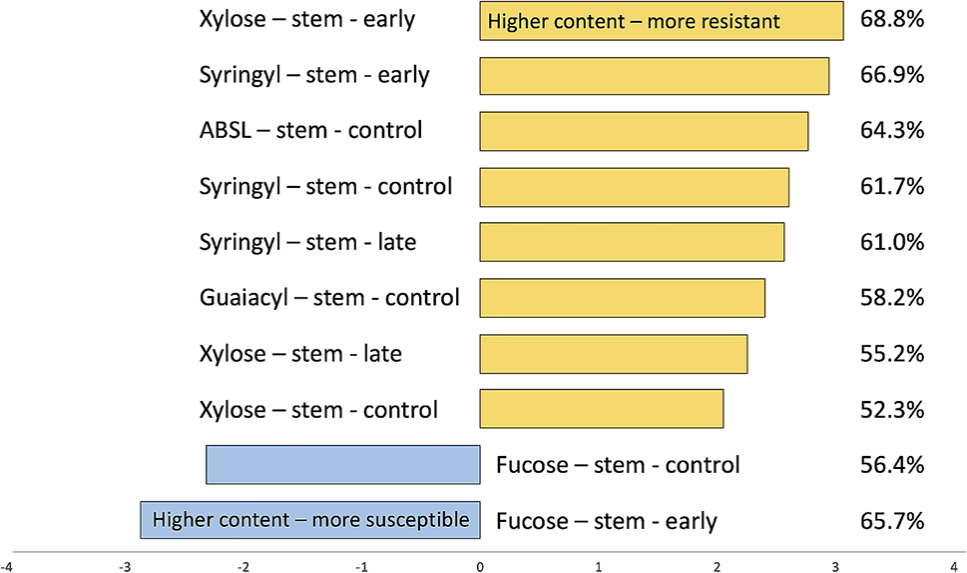
Compounds significantly correlating with resistance to *S. minor*. AUDPS scores of disease progression were correlated with cell walls composition, and those significant at *p* < 0.05 after false discovery rate adjustment are shown. Values on the right show percent of phenotypic variation in AUDPS explained by the respective compound content

Correlation analysis between disease progression (AUDPS values) and cell wall composition revealed significant (*p* ≤ 0.05) associations in stems for fucose, xylose, ABSL, guaiacyl, and syringyl at different disease progression stages (Fig. 5). Higher levels of xylose, ABSL, guaiacyl, and syringyl were associated with slower disease progression, while higher levels of fucose were associated with more rapid disease progression.

**Fig. 6:**
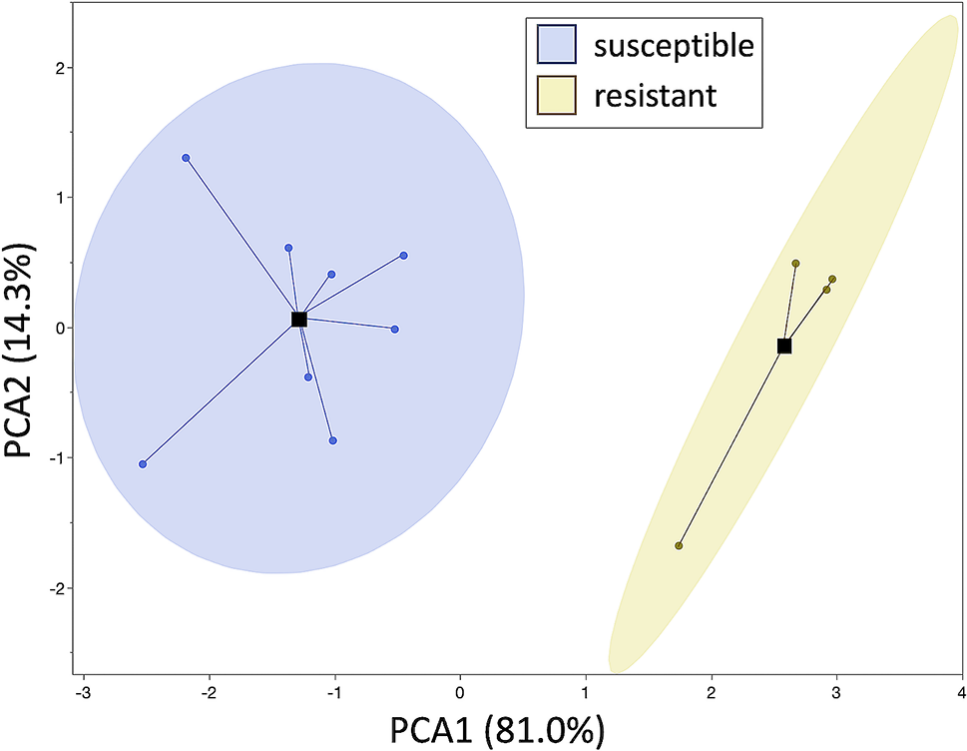
Principal component analysis based on ABSL, syringyl, guaiacyl, xylose, and fucose content in stem cell walls of healthy, uninoculated plants. The four compounds were selected based on the correlation analysis results shown in Fig. 5. Note that the grouping of plants into two groups is identical to that for disease progression and stem mechanical strength (Fig. S1)

Since composition data from uninoculated, control plants hold the most practical use for plant breeders, the content of ABSL, syringyl, guaiacyl, xylose, and fucose was used in principal component analysis. The PCA results using composition data from healthy plants (Fig. 6) closely resembled results obtained from disease resistance and stem strength data (Fig. S1), indicating a close match between the results.

**Fig. 7:**
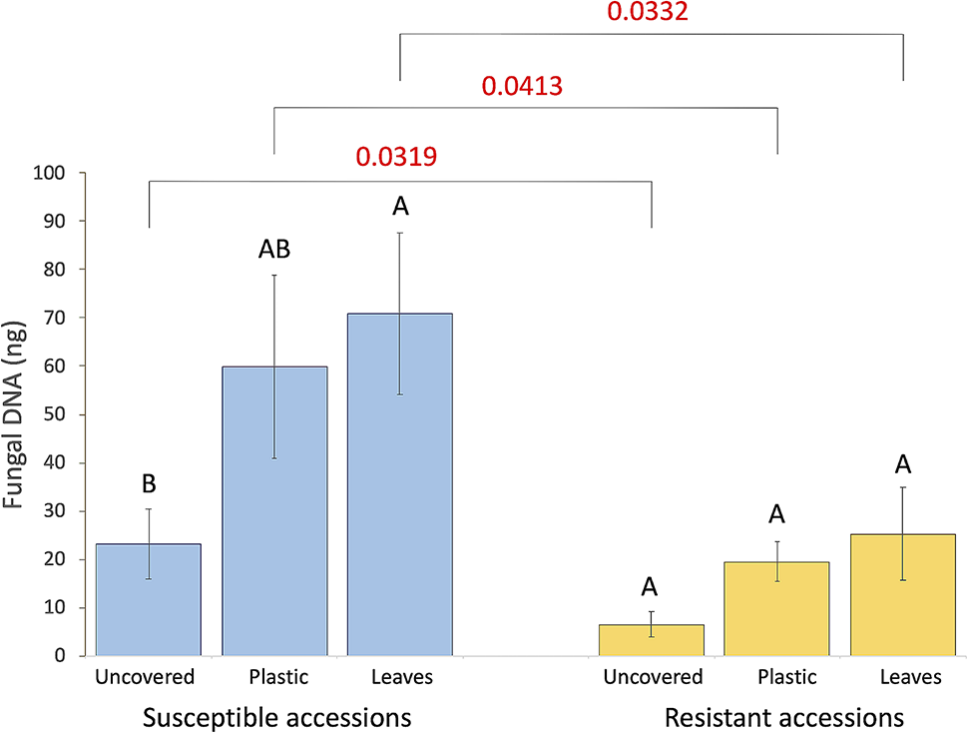
Fungal biomass accumulation in susceptible and resistant accessions under three treatments. After inoculation with *S. minor* mycelia, basal stems were either left uncovered (‘uncovered’ group), wrapped with the plant’s own leaves (‘leaves’ group), or wrapped with clear plastic wrap (‘plastic’ group). Mean values within each group, followed by different letters, are significantly different (*p* ≤ 0.05) based on Tukey’s HSD test. *P*-values at the top show differences between identical treatments on the two groups of accessions

Quantification of fungal DNA showed that susceptible accessions consistently had higher amounts of *S. minor* DNA than resistant accessions across all treatments (Fig. 7). The effect size was significant for the phenotypic groups (susceptible vs. resistant, *η*^2^ = 0.185, *p* < 0.001) and microenvironmental conditions (wrapped vs. uncovered, *η*^2^ = 0.065, *p* < 0.05), but not for additional nutrient availability (leaves vs. no leaves, *η*^2^ = 0.008).

## Discussion

Several hundred lettuce accessions were tested for resistance to *S. minor*, with substantial resistance observed in the oil-seed accession PI 251246 [13, 17, 55]. Contrary to initial hypotheses of resistance mechanism being related to pathogen avoidance [12], recent studies suggest that resistance in PI 251246 and 11-G99 is linked to stem mechanical strength rather than pathogen avoidance [14]. In this study, we investigated the difference in stem and root composition between accessions with rapid basal stem degradation (Da Ye Wo Sun, Eruption, Reine des Glaces, and Salinas) and slow basal stem degradation (11-G99 and PI 251246) following inoculations with *S. minor* under greenhouse conditions.

We found that syringyl and guaiacyl play vital roles in lettuce constitutive resistance to *S. minor*, as both lignin monomers were significantly higher in cell walls of stems of healthy plants from resistant accessions compared to susceptible accessions (Figs. 3 and 5). This finding is consistent with previous studies that demonstrated stem cell wall lignification enhances host resistance to *Sclerotinia* spp. [40, 56–60]. Additionally, lignification plays a role in induced components of lettuce resistance, as *de novo* synthesis of both lignin monomers in stems and roots occurred after inoculation with the pathogen (Fig. 1). However, some previous studies suggested lower stem lignin content may be linked to higher resistance against *Sclerotinia* spp. [44, 46], while also concluding that stem lignin content alone is not a reliable indicator of resistance [45]. Therefore, it has been proposed that rather than just the total lignin content, lignin intermediates such as caffeic and ferulic acids may play a significant role in the resistance response [47].

While previous studies suggested a decreasing syringyl to guaiacyl (S/G) ratio is associated with improved resistance to *Sclerotinia* spp. [40, 56, 60], our results did not show a clear relationship between the S/G ratio and resistance. The S/G ratio was higher in the stem cell walls of healthy resistant plants (2.26) than in susceptible plants before inoculation (1.61). However, this ratio slightly but consistently decreased in both resistant (2.18) and susceptible (1.43) accessions at the late stage of disease progression. Thus, it is possible that lignin cross-linking, rather than its composition, is directly related to lettuce drop resistance, as syringyl, guaiacyl, and hydroxyphenyl have similar inhibitory effects on cell wall degradability by fungal hydrolases [61, 62]. Additionally, not only the absolute amount of syringyl and guaiacyl or their S/G ratio may influence resistance, but also the location of their deposition. For example, in moderately resistant cultivar of *B. napus* infected by *S. sclerotiorum*, syringyl deposition occurred earlier than in susceptible cultivar [57]. Syringyl lignification was observed in the vascular sclerenchymatic cortex tissue, while guaiacyl was detected in both resistant and susceptible cultivars in the intercellular spaces of sclereids and adjacent phloem cells. Thus, earlier deposition of syringyl appears to be related to the infection response, whereas guaiacyl deposition, which was similar in both resistant and susceptible cultivars, is possibly a part of basal plant defense [57].

Among the tested monosaccharides, xylose and fucose content in stem cell walls showed the most consistent results, with higher levels of xylose and lower levels of fucose associated with increased resistance (Figs. 2, 3 and 5). Xylose, the main component of hemicellulose, has been implicated in increased resistance of transgenic *B. napus* plants to *S. sclerotiorum* [63] and hypothesized to be one of the determinants controlling *Arabidopsis thaliana* resistance to another necrotrophic fungus, *Plectosphaerella cucumerina* [64].

The association of higher fucose content in lettuce with increased susceptibility to *S. minor* is unexpected, as fucose typically enhances plant resistance by aiding cell wall xyloglucan biosynthesis. For example, *A. thaliana* mutants with higher levels of fucosylated xyloglucans show greater resistance to the necrotrophic fungus *P. cucumerina* [65]. We speculate that the increased fucose in susceptible lettuce may relate to fucosylated polysaccharides in the primary cell wall, serving as targets for pathogen lectins [66]. Proteins structurally similar to fucose-specific lectins have been found in *S. sclerotiorum* [67, 68]. The interaction between fucosylated xyloglucans in lettuce cell walls and pathogen lectins might facilitate pathogen recognition and infection, explaining the unexpected link between higher fucose content and increased susceptibility to *S. minor*.

Similar patterns to that of fucose content were observed for arabinose, galactose, and rhamnose content in stem cell walls (Fig. 2), though the results were not consistently significant. Initially these monosaccharides were higher in susceptible accessions but gradually decreased in both susceptible and resistant accessions as the disease progressed. Sclerotiniaceae lectins have binding specificity towards all these compounds [69].

Conversely, as the disease developed, the glucose content increased, particularly in susceptible accessions (Fig. 4). Glucose molecules are utilized to produce cellulose and can be incorporated into other cell wall components such as hemicelluloses and pectins. *Sclerotinia* spp. can alter the composition of plant cell walls, including the breakdown of polysaccharides like cellulose and hemicellulose, which may release glucose.

Although the relationship between root composition and resistance was not consistent, root cell wall composition may contribute to the plant’s response to *S. minor* infection. Compounds such as syringyl and guaiacyl (Fig. 3) showed a pattern similar to that observed in stems. Interestingly, like in stems, the glucose levels in roots of susceptible accessions substantially increased as the disease progressed from 9.5 µg/mg to 16.9 µg/mg to 37.2 µg/mg (Fig. 4), suggesting a potential relationship between glucose levels in cell walls of both stems and roots and resistance to basal stem degradation caused by *S. minor*.

Our results indicate that enhancing lettuce’s resistance to *S. minor* could be improved through increased levels of lignin and/or hemicellulose in host tissues. However, it is crucial to implement these changes carefully to prevent negative impacts on plant growth, leaf texture, digestibility, taste, and nutritional value. Therefore, any improvement in cell wall mechanical strength via elevated lignocellulosic compound levels should be specifically targeted to the basal stem. If achieving these modifications naturally proves challenging, tissue-specific gene expression approaches may be considered [70, 71].

It is important to note that cultivar Eruption, previously identified as one of the most resistant accessions to *S. minor* in field trials [11, 13, 55, 72], exhibited elevated disease severity (rapid basal stem degradation) when inoculated with *S. minor* in a greenhouse. This difference in resistance may be related to the anthocyanin content, which decreases under greenhouse conditions compared to field conditions, mostly due to lower ultraviolet (UV) radiation [73]. Loci regulating lettuce anthocyanin content and resistance to *S. minor* frequently co-locate [55], indicating a possible involvement of anthocyanins in resistance to *Sclerotinia* spp. [74]. In soybean, genes encoding for enzymes involved in anthocyanin and phytoalexin biosynthesis were upregulated in resistant plants [47]. Anthocyanins may function as inhibitors of fungal growth [75] and as an antioxidants by scavenging reactive oxygen species (ROS), thereby limiting the induction of plant cell death [76]. Alternatively, resistance to *S. minor* in cultivar Eruption could be based on *R*-gene(s), but their expression is modulated by environmental conditions prior to infection [77, 78]. It is also possible that cv. Eruption has a mechanism allowing avoidance of natural *S. minor* infection in the field.

Quantification of fungal DNA revealed that microenvironmental conditions around the stem base substantially influence disease progression and pathogen development. The lowest quantities of *S. minor* DNA were detected in stems with uncovered bases (Fig. 7). This aligns with previous findings that lettuce plants exhibit higher disease severity in the field under higher relative humidity [14], as humidity is expected to be higher when stem bases are wrapped. In field conditions, when relative humidity ranged from 65 to 95% and temperature from 11 °C to 18 °C near the lettuce stem base, the highest disease rating occurred at temperatures below 11.5 °C or relative humidity above 90% [14]. Small but consistent differences between wrapping stem bases in clear plastic or the plant’s own leaves suggest that leaves may also provide additional nutrients to the pathogen. Although our simplified effect size model does not account for all environmental or biological factors, it indicates that creating microenvironmental conditions less hospitable for pathogen proliferation could reduce or delay lettuce drop symptoms in lettuce plants.

## Conclusions

Our results suggest that stem cell wall lignification and elevated levels of hemicellulose, as indicated by xylose and arabinose content, contribute to slower degradation of the basal stem after infection with *S. minor*. However, further experiments with larger plant sets are needed to determine the individual effect of each compound and confirm the involvement of fucose in the lettuce-*S. minor* interaction. Future studies will focus on detailed analyses of stem physical properties and cell wall composition in recombinant inbred lines from the Salinas × PI 251246 mapping population, which exhibit considerable variation in these traits and resistance to lettuce drop [12]. These analyses will enhance our understanding of the relationship between resistance to basal stem degradation caused by *S. minor*, stem strength, and cell wall composition. By analyzing mapped quantitative trait loci (QTL) and their positions on the reference genome, we can identify candidate genes involved in resistance, including those in the lignin, hemicellulose, and anthocyanidin biosynthesis pathways. Subsequent steps may include functional analysis of these key genes to elucidate their specific roles in resistance mechanisms.

Our results indicate that targeting hard, lignified stems could allow for the development of lettuce lines with more durable resistance to lettuce drop. The content of lignin, particularly guaiacyl and syringyl, and xylose could serve as biomarkers for selecting lines with enhanced resistance. It is important to reiterate that any increase in lignin content would need to be localized in stems (or even the stem base) and not the leaf tissue consumed. Additionally, improvements in resistance may be achieved by developing lines with a microenvironment around the stem base that is less hospitable for pathogen proliferation.

### Electronic supplementary material

Below is the link to the electronic supplementary material.

Supplementary Material 1

## Data Availability

All data generated or analyzed during this study are included in this published article and its supplementary information files.

## Abbreviations

ABSL: Acetyl bromide soluble lignin
ANOVA: Analysis of variance
AUDPS: Area under the disease progress stairs
CDS: Coding sequence
Ct: Cycle threshold
CWDEs: Cell wall-degrading enzymes
EDTA: Ethylenediaminetetraacetic acid
FDR: False discovery rate
HSD test: Honestly significant difference test
PAMR: Plant architecture- or stem strength-mediated resistance
PCA: Principal component analysis
qPCR: Quantitative polymerase chain reaction
SDS: Sodium dodecyl sulfate
S/G ratio: Syringyl to guaiacyl ratio
TE buffer: Tris-EDTA buffer

## References

[1] Barrière V, Lecompte F, Nicot PC, Maisonneuve B, Tchamitchian M, Lescourret F. Lettuce cropping with less pesticides. A review. Agron Sustain Dev. 2014;34:175–98. 10.1007/s13593-013-0158-5

[2] Boland GJ, Hall R. Index of plant hosts of *Sclerotinia Sclerotiorum*. Can J Plant Pathol. 1994;16(2):93–108. 10.1080/07060669409500766

[3] Farr DF, Rossman AY. Fungal Databases. US National Fungus Collections, ARS, USDA. 2020; https://nt.ars-grin.gov/fungaldatabases/ Retrieved June 15, 2020.

[4] Fisher MC, Gurr SJ, Cuomo CA, Blehert DS, Jin H, Stukenbrock EH, Stajich JE, Kahmann R, Boone C, Denning DW. Threats posed by the fungal kingdom to humans, wildlife, and agriculture. mBio. 2020;11(3):e00449–20. 32371596 10.1128/mBio.00449-20 PMC7403777

[5] Isnaini M, Keane PJ. Biocontrol and epidemiology of lettuce drop caused by *Sclerotinia minor* at Bacchus Marsh, Victoria. Australas Plant Pathol. 2007;36:295–304. 10.1071/AP07024

[6] Subbarao KV. Progress toward integrated management of lettuce drop. Plant Dis. 1998;82(10):1068–78. 30856764 10.1094/PDIS.1998.82.10.1068

[7] Steadman JR. Control of plant diseases caused by *Sclerotinia* species. Phytopathology. 1979;69(8):904–7. 10.1094/Phyto-69-904

[8] Adams PB, Ayers WA. Ecology of *Sclerotinia* species. Phytopathology. 1979;69(8):896–9. 10.1094/Phyto-69-896

[9] Melzer MS, Smith EA, Boland GJ. Survey of lettuce drop at Holland Marsh, Ontario. Can Plant Disease Surv. 1993;73:105.

[10] Cessna SG, Sears VE, Dickman MB, Low PS. Oxalic acid, a pathogenicity factor for *Sclerotinia Sclerotiorum*, suppresses the oxidative burst of the host plant. Plant Cell. 2000;12(11):2191–9. 11090218 10.1105/tpc.12.11.2191 PMC150167

[11] Mamo BE, Hayes RJ, Truco MJ, Puri KD, Michelmore RW, Subbarao KV, Simko I. The genetics of resistance to lettuce drop (*Sclerotinia* spp.) in lettuce in a recombinant inbred line population from Reine Des Glaces × Eruption. Theor Appl Genet. 2019;132:2439–60. 31165222 10.1007/s00122-019-03365-6

[12] Grube RC. Genetic analysis of resistance to lettuce drop caused by *Sclerotinia minor*. Acta Hort. 2004;637:49–55. 10.17660/ActaHortic.2004.637.4

[13] Hayes RJ, Wu BM, Pryor BM, Chitrampalam P, Subbarao KV. Assessment of resistance in lettuce (Lactuca sativa L.) to mycelial and ascospore infection by Sclerotinia minor Jagger and S. Sclerotiorum (Lib.) De Bary. HortScience. 2010;45(3):333–41. 10.21273/HORTSCI.45.3.333

[14] Mamo BE, Eriksen RL, Adhikari ND, Hayes RJ, Mou B, Simko I. Epidemiological characterization of lettuce drop and biophysical features of the host identify soft stem as a susceptibility factor to *Sclerotinia minor*. PhytoFrontiers. 2021;1:182–204. 10.1094/PHYTOFR-12-20-0040-R

[15] Newton HC, Sequeira L. Possible sources of resistance in lettuce to *Sclerotinia Sclerotiorum*. Plant Disease Report. 1972;56:875–8.

[16] Chen Z, Han Y, Ning K, Ding Y, Zhao W, Yan S, Luo C, Jiang X, Ge D, Liu R. Inflorescence development and the role of LsFT in regulating bolting in lettuce (*Lactuca sativa* L). Front Plant Sci. 2018;8:2248. 29403510 10.3389/fpls.2017.02248 PMC5778503

[17] Grube R, Ryder E. Identification of lettuce (*Lactuca sativa* L.) germplasm with genetic resistance to drop caused by *Sclerotinia minor*. J Am Soc Hortic Sci. 2004;129(1):70–6. 10.21273/JASHS.129.1.0070

[18] Rosental L, Still DW, You Y, Hayes RJ, Simko I. Mapping and identification of genetic loci affecting earliness of bolting and flowering in lettuce. Theor Appl Genet. 2021;134:3319–37. 34196730 10.1007/s00122-021-03898-9

[19] Ryder EJ, Milligan DC. Additional genes controlling flowering time in *Lactuca sativa* and *L. Serriola*. J Am Soc Hortic Sci. 2005;130(3):448–53. 10.21273/JASHS.130.3.448

[20] McCaghey M, Willbur J, Smith DL, Kabbage M. The complexity of the *Sclerotinia sclerotiorum* pathosystem in soybean: virulence factors, resistance mechanisms, and their exploitation to control Sclerotinia stem rot. Trop Plant Pathol. 2019;44:12–22. 10.1007/s40858-018-0259-4

[21] Wang Z, Ma L-Y, Cao J, Li Y-L, Ding L-N, Zhu K-M, Yang Y-H, Tan X-L. Recent advances in mechanisms of plant defense to *Sclerotinia Sclerotiorum*. Front Plant Sci. 2019;10:1314. 31681392 10.3389/fpls.2019.01314 PMC6813280

[22] Amselem J, Cuomo CA, van Kan JAL, Viaud M, Benito EP, Couloux A, Coutinho PM, de Vries RP, Dyer PS, Fillinger S. Genomic analysis of the necrotrophic fungal pathogens *Sclerotinia sclerotiorum* and *Botrytis Cinerea*. PLoS Genet. 2011;7(8):e1002230. 21876677 10.1371/journal.pgen.1002230 PMC3158057

[23] Liang X, Rollins JA. Mechanisms of broad host range necrotrophic pathogenesis in *Sclerotinia Sclerotiorum*. Phytopathology. 2018;108(10):1128–40. 30048598 10.1094/PHYTO-06-18-0197-RVW

[24] Wang M, Zhu X, Wang KE, Lu C, Luo M, Shan T, Zhang Z. A wheat caffeic acid 3-O-methyltransferase TaCOMT-3D positively contributes to both resistance to sharp eyespot disease and stem mechanical strength. Sci Rep. 2018;8(1):6543. 29695751 10.1038/s41598-018-24884-0 PMC5916939

[25] Xiang D, Song Y, Wu Q, Ma C, Zhao J, Wan Y, Zhao G. Relationship between stem characteristics and lodging resistance of Tartary buckwheat (*Fagopyrum tataricum*). Plant Prod Sci. 2019;22(2):202–10. 10.1080/1343943X.2019.1577143

[26] Denton-Giles M, Derbyshire MC, Khentry Y, Buchwaldt L, Kamphuis LG. Partial stem resistance in *Brassica napus* to highly aggressive and genetically diverse *Sclerotinia sclerotiorum* isolates from Australia. Can J Plant Pathol. 2018;40(4):551–61. 10.1080/07060661.2018.1516699

[27] McCaghey M, Willbur J, Ranjan A, Grau CR, Chapman S, Diers B, Groves C, Kabbage M, Smith DL. Development and evaluation of *Glycine max* germplasm lines with quantitative resistance to *Sclerotinia Sclerotiorum*. Front Plant Sci. 2017;8:1495. 28912790 10.3389/fpls.2017.01495 PMC5584390

[28] Zhao Q, Dixon RA. Altering the cell wall and its impact on plant disease: from forage to bioenergy. Annu Rev Phytopathol. 2014;52:69–91. 24821183 10.1146/annurev-phyto-082712-102237

[29] Amsbury S, Kirk P, Benitez-Alfonso Y. Emerging models on the regulation of intercellular transport by plasmodesmata-associated callose. J Exp Bot. 2018;69(1):105–15. 10.1093/jxb/erx337 29040641

[30] Cheong YH, Chang H-S, Gupta R, Wang X, Zhu T, Luan S. Transcriptional profiling reveals novel interactions between wounding, pathogen, abiotic stress, and hormonal responses in Arabidopsis. Plant Physiol. 2002;129(2):661–77. 12068110 10.1104/pp.002857 PMC161692

[31] Hamann T. Plant cell wall integrity maintenance as an essential component of biotic stress response mechanisms. Front Plant Sci. 2012;3:77. 22629279 10.3389/fpls.2012.00077 PMC3355559

[32] Le Gall H, Philippe F, Domon J-M, Gillet F, Pelloux J, Rayon C. Cell wall metabolism in response to abiotic stress. Plants. 2015;4(1):112–66. 27135320 10.3390/plants4010112 PMC4844334

[33] Jia X-L, Wang G-L, Xiong F, Yu X-R, Xu Z-S, Wang F, Xiong A-S. De novo assembly, transcriptome characterization, lignin accumulation and anatomic characteristics: novel insights into lignin biosynthesis during celery leaf development. Sci Rep. 2015;5(1):8259. 25651889 10.1038/srep08259 PMC4317703

[34] Trabucco GM, Matos DA, Lee SJ, Saathoff AJ, Priest HD, Mockler TC, Sarath G, Hazen SP. Functional characterization of cinnamyl alcohol dehydrogenase and caffeic acid O-methyltransferase in *Brachypodium distachyon*. BMC Biotechnol. 2013;13(1):61. 23902793 10.1186/1472-6750-13-61 PMC3734214

[35] Bari E, Mohebby B, Naji HR, Oladi R, Yilgor N, Nazarnezhad N, Ohno KM, Nicholas DD. Monitoring the cell wall characteristics of degraded beech wood by white-rot fungi: anatomical, chemical, and photochemical study. Maderas Ciencia Y tecnología. 2018;20(1):35–56.

[36] Skyba O, Douglas CJ, Mansfield SD. Syringyl-rich lignin renders poplars more resistant to degradation by wood decay fungi. Appl Environ Microbiol. 2013;79(8):2560–71. 23396333 10.1128/AEM.03182-12 PMC3623167

[37] Bellincampi D, Cervone F, Lionetti V. Plant cell wall dynamics and wall-related susceptibility in plant–pathogen interactions. Front Plant Sci. 2014;5:228. 24904623 10.3389/fpls.2014.00228 PMC4036129

[38] Sattler SE, Funnell-Harris DL. Modifying lignin to improve bioenergy feedstocks: strengthening the barrier against pathogens? Front Plant Sci. 2013;4:70. 23577013 10.3389/fpls.2013.00070 PMC3617363

[39] Yang X, Wang H, Lui G, Wang X. The lignin biosynthesis regulation and its relationship with Sclerotinia and lodging resistances for Brassica napus. 12th Int Rape Seed Congress Wuhan. 2007;2:50–2.

[40] Eynck C, Séguin-Swartz G, Clarke WE, Parkin IAP. Monolignol biosynthesis is associated with resistance to *Sclerotinia sclerotiorum* in *Camelina sativa*. Mol Plant Pathol. 2012;13(8):887–99. 22487550 10.1111/j.1364-3703.2012.00798.x PMC6638904

[41] Zhao J, Buchwaldt L, Rimmer SR, Sharpe A, McGregor L, Bekkaoui D, Hegedus D. Patterns of differential gene expression in *Brassica napus* cultivars infected with *Sclerotinia Sclerotiorum*. Mol Plant Pathol. 2009;10(5):635–49. 19694954 10.1111/j.1364-3703.2009.00558.x PMC6640428

[42] Wei L, Jian H, Lu K, Yin N, Wang J, Duan X, Li W, Liu L, Xu X, Wang R. Genetic and transcriptomic analyses of lignin-and lodging-related traits in *Brassica napus*. Theor Appl Genet. 2017;130:1961–73. 28634809 10.1007/s00122-017-2937-x

[43] Ding Y, Mei J, Chai Y, Yu Y, Shao C, Wu Q, Disi JO, Li Y, Wan H, Qian W. Simultaneous transcriptome analysis of host and pathogen highlights the interaction between *Brassica oleracea* and *Sclerotinia Sclerotiorum*. Phytopathology. 2019;109(4):542–50. 30265202 10.1094/PHYTO-06-18-0204-R

[44] Peltier AJ, Hatfield RD, Grau CR. Soybean stem lignin concentration relates to resistance to *Sclerotinia Sclerotiorum*. Plant Dis. 2009;93(2):149–54. 30764097 10.1094/PDIS-93-2-0149

[45] Bennett RS, Hatfield RD, Payton ME, Chamberlin KD. Lignin content and resistance to *Sclerotinia minor* in peanut. Peanut Sci. 2017;44(1):35–41. 10.3146/PS16-10.1

[46] Jiang J, Liao X, Jin X, Tan L, Lu Q, Yuan C, Xue Y, Yin N, Lin N, Chai Y. MYB43 in oilseed rape (*Brassica napus*) positively regulates vascular lignification, plant morphology and yield potential but negatively affects resistance to *Sclerotinia Sclerotiorum*. Genes. 2020;11(5):581. 32455973 10.3390/genes11050581 PMC7290928

[47] Ranjan A, Westrick NM, Jain S, Piotrowski JS, Ranjan M, Kessens R, Stiegman L, Grau CR, Conley SP, Smith DL. Resistance against *Sclerotinia sclerotiorum* in soybean involves a reprogramming of the phenylpropanoid pathway and up-regulation of antifungal activity targeting ergosterol biosynthesis. Plant Biotechnol J. 2019;17(8):1567–81. 30672092 10.1111/pbi.13082 PMC6662107

[48] Santoro N, Cantu SL, Tornqvist C-E, Falbel TG, Bolivar JL, Patterson SE, Pauly M, Walton JD. A high-throughput platform for screening milligram quantities of plant biomass for lignocellulose digestibility. Bioenergy Res. 2010;3:93–102. 10.1007/s12155-009-9074-6

[49] Foster CE, Martin TM, Pauly M. Comprehensive compositional analysis of plant cell walls (lignocellulosic biomass) part I: Lignin. JoVE (Journal Visualized Experiments). 2010(37):e1745. 10.3791/1745 PMC3144576 20224547

[50] Foster CE, Martin TM, Pauly M. Comprehensive compositional analysis of plant cell walls (lignocellulosic biomass) part II: Carbohydrates. JoVE (Journal Visualized Experiments). 2010(37):e1837. 10.3791/1837 PMC3145335 20228730

[51] Harman-Ware AE, Foster C, Happs RM, Doeppke C, Meunier K, Gehan J, Yue F, Lu F, Davis MF. A thioacidolysis method tailored for higher‐throughput quantitative analysis of lignin monomers. Biotechnol J. 2016;11(10):1268–73. 27534715 10.1002/biot.201600266 PMC5096032

[52] Klosterman SJ. Real-time PCR for the quantification of fungi in planta. In: Bolton M, Thomma B, editors. Plant Fungal pathogens methods and protocols. Volume 835. New York, NY: Humana; 2012. pp. 121–32. 10.1007/978-1-61779-501-5_8 22183651

[53] Green MR, Sambrook J. Molecular cloning: a laboratory manual. 4th ed. Cold Spring Harbor, NY: Cold Spring Harbor Laboratory Press; 2012.

[54] Simko I, Piepho H-P. The area under the disease progress stairs: calculation, advantage, and application. Phytopathology. 2012;102(4):381–9. 22122266 10.1094/PHYTO-07-11-0216

[55] Simko I, Sthapit Kandel J, Peng H, Zhao R, Subbarao KV. Genetic determinants of lettuce resistance to drop caused by *Sclerotinia minor* identified through genome-wide association mapping frequently co-locate with loci regulating anthocyanin content. Theor Appl Genet. 2023;136:180. 37548768 10.1007/s00122-023-04421-y

[56] Cao Y, Yan X, Ran S, Ralph J, Smith RA, Chen X, Qu C, Li J, Liu L. Knockout of the lignin pathway gene BnF5H decreases the S/G lignin compositional ratio and improves *Sclerotinia sclerotiorum* resistance in *Brassica napus*. Plant, Cell & Environment. 2022;45(1):248 – 61. 10.1111/pce.14208 PMC9084453 34697825

[57] Höch K, Koopmann B, von Tiedemann A. Lignin composition and timing of cell wall lignification are involved in *Brassica napus* resistance to stem rot caused by *Sclerotinia Sclerotiorum*. Phytopathology. 2021;111(8):1438–48. 33386067 10.1094/PHYTO-09-20-0425-R

[58] Liu D, Wu J, Lin L, Li P, Li S, Wang Y, Li J, Sun Q, Liang J, Wang Y. Overexpression of Cinnamoyl-CoA Reductase 2 in *Brassica napus* increases resistance to *Sclerotinia sclerotiorum* by affecting lignin biosynthesis. Front Plant Sci. 2021;12:732733. 34630482 10.3389/fpls.2021.732733 PMC8494948

[59] Uloth MB, Clode PL, You MP, Barbetti MJ. Attack modes and defence reactions in pathosystems involving Sclerotinia Sclerotiorum, Brassica carinata, B. Juncea and B. Napus. Ann Botany. 2016;117(1):79–95. 26420204 10.1093/aob/mcv150 PMC4701150

[60] Shao Y, Shen Y, He F, Li Z. QTL identification for stem fiber, strength and rot resistance in a DH population from an alien introgression of *Brassica napus*. Plants. 2022;11:373. 35161354 10.3390/plants11030373 PMC8840419

[61] Grabber JH. How do lignin composition, structure, and cross-linking affect degradability? A review of cell wall model studies. Crop Sci. 2005;45(3):820–31. 10.2135/cropsci2004.0191

[62] Grabber JH, Ralph J, Hatfield RD, Quideau S. -Hydroxyphenyl, guaiacyl, and syringyl lignins have similar inhibitory effects on wall degradability. J Agric Food Chem. 1997;45(7):2530–2. 10.1021/jf970029v

[63] Wang Z, Wan L, Xin Q, Chen Y, Zhang X, Dong F, Hong D, Yang G. Overexpression of OsPGIP2 confers *Sclerotinia sclerotiorum* resistance in *Brassica napus* through increased activation of defense mechanisms. J Exp Bot. 2018;69(12):3141–55. 29648614 10.1093/jxb/ery138 PMC5972623

[64] Delgado-Cerezo M, Sánchez-Rodríguez C, Escudero V, Miedes E, Fernández PV, Jordá L, Hernández-Blanco C, Sánchez-Vallet A, Bednarek P, Schulze-Lefert P, et al. *Arabidopsis* heterotrimeric G-protein regulates cell wall defense and resistance to necrotrophic fungi. Mol Plant. 2012;5(1):98–114. 21980142 10.1093/mp/ssr082

[65] Molina A, Miedes E, Bacete L, Rodríguez T, Mélida H, Denancé N, Sánchez-Vallet A, Rivière M-P, López G, Freydier A. *Arabidopsis* cell wall composition determines disease resistance specificity and fitness. Proc Natl Acad Sci USA. 2021;118(5):e2010243118. 33509925 10.1073/pnas.2010243118 PMC7865177

[66] Kostlánová N, Mitchell EP, Lortat-Jacob H, Oscarson S, Lahmann M, Gilboa-Garber N, Chambat G, Wimmerová M, Imberty A. The fucose-binding lectin from *Ralstonia solanacearum*: a new type of β-propeller architecture formed by oligomerization and interacting with fucoside, fucosyllactose, and plant xyloglucan. J Biol Chem. 2005;280(30):27839–49. 15923179 10.1074/jbc.M505184200

[67] Candy L, Van Damme EJ, Peumans WJ, Menu-Bouaouiche L, Erard M, Rougé P. Structural and functional characterization of the GalNAc/Gal-specific lectin from the phytopathogenic ascomycete *Sclerotinia Sclerotiorum* (Lib.) De Bary. Biochem Biophys Res Commun. 2003;308(2):396–402. 12901882 10.1016/S0006-291X(03)01406-2

[68] Li M, Rollins JA. The development-specific *ssp1* and *ssp2* genes of *Sclerotinia Sclerotiorum* encode lectins with distinct yet compensatory regulation. Fungal Genet Biol. 2010;47(6):531–8. 20350614 10.1016/j.fgb.2010.03.008

[69] Kellens JT, Goldstein IJ, Peumans WJ. Lectins in different members of the Sclerotiniaceae. Mycol Res. 1992;96(6):495–502. 10.1016/S0953-7562(09)81097-6

[70] Berthet S, Demont-Caulet N, Pollet B, Bidzinski P, Cézard L, Le Bris P, Borrega N, Hervé J, Blondet E, Balzergue S, et al. Disruption of *LACCASE4* and *17* results in tissue-specific alterations to lignification of *Arabidopsis thaliana* stems. Plant Cell. 2011;23(3):1124–37. 21447792 10.1105/tpc.110.082792 PMC3082258

[71] Kumar M, Campbell L, Turner S. Secondary cell walls: biosynthesis and manipulation. J Exp Bot. 2016;67(2):515–31. 26663392 10.1093/jxb/erv533

[72] Simko I, Subbarao KV, Hayes R. Breeding lettuce for resistance against *Sclerotinia minor*. HortScience. 2023;58(12):1526–32. 10.21273/HORTSCI17399-23

[73] Sytar O, Zivcak M, Bruckova K, Brestic M, Hemmerich I, Rauh C, Simko I. Shift in accumulation of flavonoids and phenolic acids in lettuce attributable to changes in ultraviolet radiation and temperature. Sci Hort. 2018;239:193–204. 10.1016/j.scienta.2018.05.020

[74] Liu R, Ding L-N, Li M, Cao W, Wang Y-K, Wang W-J, Yu Y-K, Wang Z, Zhu K-M, Tan X-L. Characterization of a rapeseed anthocyanin-more mutant with enhanced resistance to *Sclerotinia Sclerotiorum*. J Plant Growth Regul. 2020;39(2):703–16. 10.1007/s00344-019-10011-4

[75] Liu R, Ding L-N, Li M, Cao W, Wang Y-K, Wang W-J, Yu Y-K, Wang Z, Zhu K-M, Tan X-L. Characterization of a rapeseed anthocyanin-more mutant with enhanced resistance to *Sclerotinia Sclerotiorum*. J Plant Growth Regul. 2020;39:703–16. 10.1007/s00344-019-10011-4

[76] Calla B, Blahut-Beatty L, Koziol L, Simmonds DH, Clough SJ. Transcriptome analyses suggest a disturbance of iron homeostasis in soybean leaves during white mould disease establishment. Mol Plant Pathol. 2014;15(6):576–88. 24330102 10.1111/mpp.12113 PMC6638882

[77] MacQueen A, Bergelson J. Modulation of *R*-gene expression accross environments. J Exp Bot. 2016;67:2093–105. 26983577 10.1093/jxb/erv530 PMC4793800

[78] Zhu Y, Qian W, Hua J. Temperature modulates plant defense responses through NB-LRR proteins. PLoS Pathog. 2010;6:e1000844. 20368979 10.1371/journal.ppat.1000844 PMC2848567

