# Supplementary material for: Host cell wall composition and localized microenvironment implicated in resistance to basal stem degradation by lettuce drop (Sclerotinia minor)
Source: BMC Plant Biol. 2024 Jul 29;24:717. doi: 10.1186/s12870-024-05399-5 (PMC11285140; doi:10.1186/s12870-024-05399-5)

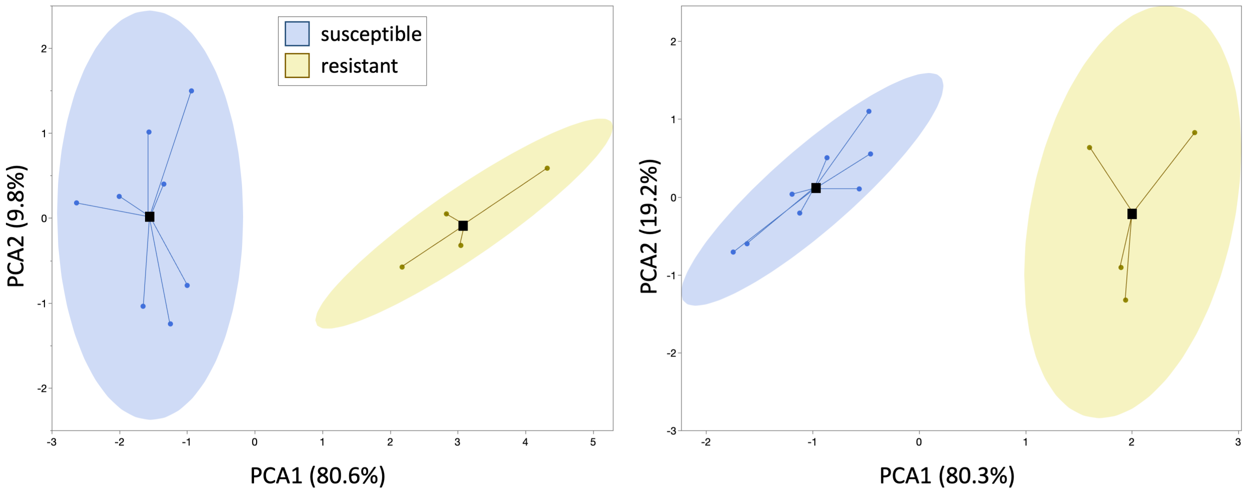

Fig. S1: Principal component analysis (PCA) based on the disease progress data (left) and the plant stem strength (right). Both analyses clearly and identically separated a group of accessions with rapid basal stem degradation caused by lettuce drop (blue color) from more resistant accessions with slower basal stem degradation (yellow color). Disease progress was evaluated after inoculation of greenhouse-grown plants with *S. minor* mycelia. PCAs were performed on data from Mamo et al. 2021 (https://doi.org/10.1094/PHYTOFR-12-20-0040-R).

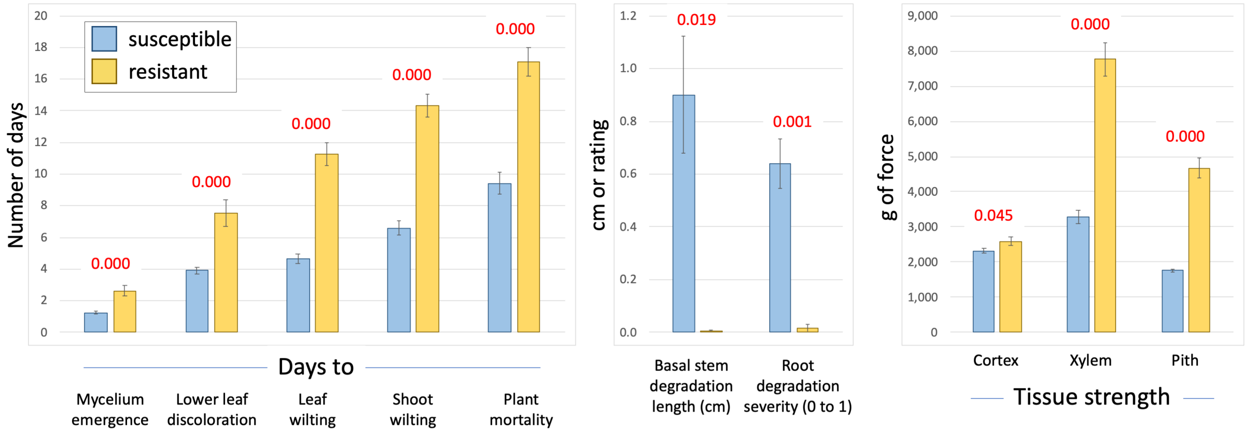

Fig. S2: Disease progress (left), stem and root degradation (center), and stem strength (right) of susceptible and partially resistant accessions. Disease progress was evaluated after inoculation of greenhouse-grown plants with *S. minor* mycelia. Numbers above bars are p-values from *t*-tests between two groups differing in resistance to *S. minor*. Values in red are significant at $p \leq 0.05$. Because p-values were rounded, 0.000 indicates $p < 0.0004$. Tests were performed on data from Mamo et al. 2021 (https://doi.org/10.1094/PHYTOFR-12-20-0040-R).

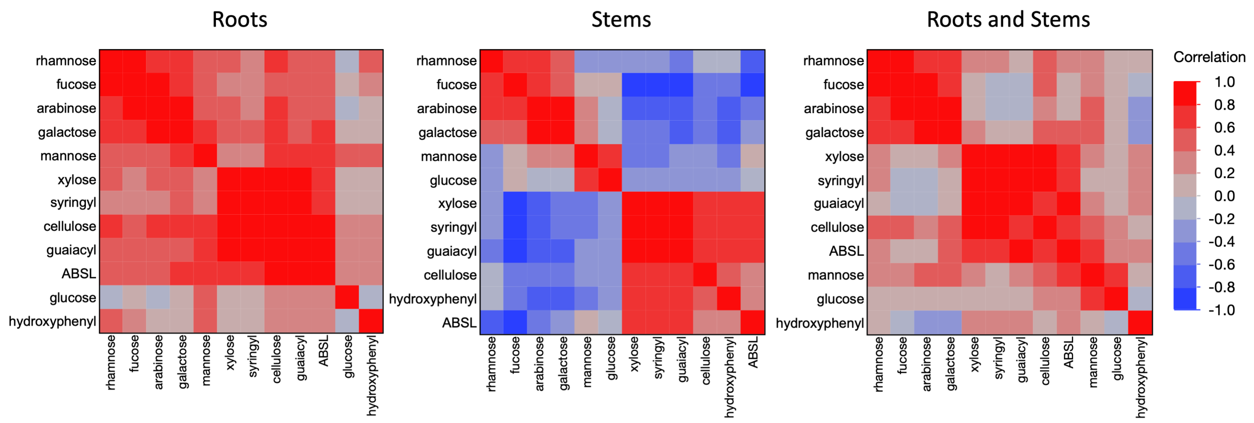

Fig. S3: Correlation coefficients between the content of 12 cell wall compounds. Correlations were calculated from root composition only (left), stem composition only (center), and combined root and stem data (right). The compounds were grouped according to their content across all accessions and disease progress stages after inoculation of greenhouse-grown plants with *S. minor* mycelia. Color indicates the magnitude of correlation coefficients, as shown on the scale located at the right-hand side.

Table S1: Statistical analyses of cell wall composition differences by ANOVA across lettuce accessions, tissue types, and disease progress.

Compound	Accession	Tissue type	Disease progress	Ac × TT	Ac × DP	TT × DP	Ac × TT × DP
**Rhamnose**	0.025	0.000	0.000	0.004	0.005	0.000	0.091
**Fucose**	0.000	0.000	0.001	0.000	0.000	0.003	0.028
**Arabinose**	0.000	0.000	0.000	0.000	0.195	0.000	0.544
**Xylose**	0.000	0.000	0.001	0.023	0.242	0.079	0.314
**Mannose**	0.428	0.000	0.043	0.538	0.534	0.089	0.127
**Galactose**	0.036	0.000	0.004	0.000	0.603	0.001	0.718
**Glucose**	0.119	0.001	0.000	0.653	0.475	0.842	0.523
**Cellulose**	0.001	0.000	0.019	0.716	0.514	0.064	0.156
**ABSL**	0.000	0.000	0.009	0.639	0.130	0.148	0.774
**Syringyl**	0.000	0.000	0.010	0.000	0.169	0.173	0.688
**Guaiacyl**	0.000	0.000	0.000	0.145	0.200	0.187	0.615
**Hydroxyphenyl**	0.001	0.426	0.001	0.287	0.039	0.235	0.695

P-values were calculated from ANOVA using six lettuce accessions (Ac), two tissue types (TT), and three disease progress (DP) stages.
Lettuce accessions: Da Ye Wo Sun, Eruption, Reine des Glaces, Salinas, PI 251246, and 11G-99
Tissue type: stems and roots
Disease progress stage: healthy (not inoculated), early stage of disease progress, and late stage of disease progress.
Because of rounding, *p*-values smaller than 0.0004 are shown as 0.000.

Table S2: Content of 12 cell wall compounds (μg/mg) averaged for six lettuce accessions, two tissue types, and three disease progression stages. Mean values within a group of accessions, tissue types, and disease progression stages followed by different letters indicate significant differences ($p \leq 0.05$, Tukey's HSD test).

Rhamnose	Content	Tukey's HSD
**Da Ye Wo Sun**	9.2	A
**Salinas**	7.7	AB
**Reine des Glaces**	7.0	AB
**11G-99**	7.0	AB
**Eruption**	6.9	AB
**PI 251246**	5.6	B
**Stems**	9.7	A
**Roots**	4.8	B
**Control (healthy, uninoculated plants)**	8.3	A
**Early stage of disease progress**	7.9	A
**Late stage of disease progress**	2.5	B

Fucose	Content	Tukey's HSD
**Da Ye Wo Sun**	1.28	A
**Salinas**	1.23	A
**Reine des Glaces**	1.11	AB
**Eruption**	1.09	AB
**11G-99**	0.91	BC
**PI 251246**	0.64	C
**Stems**	1.28	A
**Roots**	0.80	B
**Early stage of disease progress**	1.14	A
**Control (healthy, uninoculated plants)**	1.11	A
**Late stage of disease progress**	0.88	B

Arabinose	Content	Tukey's HSD
**Eruption**	19.8	A
**11G-99**	14.0	AB
**Da Ye Wo Sun**	13.5	AB
**Salinas**	13.0	B
**Reine des Glaces**	12.7	B
**PI 251246**	7.9	B

**Stems**	17.7	A
**Roots**	9.2	B

**Control (healthy, uninoculated plants)**	17.3	A
**Early stage of disease progress**	13.3	B
**Late stage of disease progress**	9.8	B

Xylose	Content	Tukey's HSD
**PI 251246**	134	A
**11G-99**	121	AB
**Da Ye Wo Sun**	97	BC
**Reine des Glaces**	67	CD
**Salinas**	60	D
**Eruption**	48	D

**Stems**	128	A
**Roots**	48	B

**Late stage of disease progress**	107	A
**Early stage of disease progress**	85	B
**Control (healthy, uninoculated plants)**	73	B

Mannose	Content	Tukey's HSD
**Da Ye Wo Sun**	7.47	A
**Eruption**	7.47	A
**11G-99**	7.41	A
**Reine des Glaces**	7.19	A
**Salinas**	6.65	A
**PI 251246**	5.71	A

**Stems**	8.19	A
**Roots**	5.77	B

**Late stage of disease progress**	7.79	A
**Early stage of disease progress**	7.17	AB
**Control (healthy, uninoculated plants)**	5.99	B

Galactose	Content	Tukey’s HSD
**Eruption**	14.4	A
**11G-99**	13.8	A
**Reine des Glaces**	12.3	A
**Da Ye Wo Sun**	10.5	A
**PI 251246**	10.2	A
**Salinas**	9.6	A

**Stems**	14.8	A
**Roots**	8.8	B

**Control (healthy, uninoculated plants)**	14.2	A
**Early stage of disease progress**	11.3	AB
**Late stage of disease progress**	9.9	B

Glucose	Content	Tukey’s HSD
**Eruption**	30.0	A
**Reine des Glaces**	28.1	A
**Da Ye Wo Sun**	27.0	A
**Salinas**	26.0	A
**11G-99**	20.0	A
**PI 251246**	16.0	A

**Stems**	30.1	A
**Roots**	18.9	B

**Late stage of disease progress**	35.0	A
**Early stage of disease progress**	22.3	B
**Control (healthy, uninoculated plants)**	16.2	B

Cellulose	Content	Tukey’s HSD
**11G-99**	299	A
**Da Ye Wo Sun**	290	A
**PI 251246**	287	AB
**Reine des Glaces**	232	ABC
**Salinas**	214	BC
**Eruption**	207	C

**Stems**	363	A
**Roots**	146	B

**Late stage of disease progress**	280	A
**Early stage of disease progress**	256	AB
**Control (healthy, uninoculated plants)**	228	B

ABSL	Content	Tukey's HSD
**PI 251246**	131	A
**11G-99**	130	A
**Da Ye Wo Sun**	113	AB
**Reine des Glaces**	107	AB
**Salinas**	95	B
**Eruption**	88	B

**Stems**	128	A
**Roots**	93	B

**Late stage of disease progress**	123	A
**Early stage of disease progress**	110	AB
**Control (healthy, uninoculated plants)**	99	B

Syringyl	Content	Tukey's HSD
**PI 251246**	32.5	A
**11G-99**	31.3	A
**Da Ye Wo Sun**	19.0	B
**Reine des Glaces**	11.9	BC
**Salinas**	11.2	BC
**Eruption**	8.1	C

**Stems**	30.3	A
**Roots**	7.7	B

**Late stage of disease progress**	22.6	A
**Early stage of disease progress**	19.4	AB
**Control (healthy, uninoculated plants)**	15.0	B

Guaiacyl	Content	Tukey's HSD
**PI 251246**	17.4	A
**11G-99**	16.4	AB
**Da Ye Wo Sun**	15.0	AB
**Reine des Glaces**	11.6	BC
**Salinas**	8.6	C
**Eruption**	6.1	C

**Stems**	15.8	A
**Roots**	9.2	B

**Late stage of disease progress**	15.8	A
**Early stage of disease progress**	12.2	B
**Control (healthy, uninoculated plants)**	9.6	B

Hydroxyphenyl	Content	Tukey's HSD
**11G-99**	0.121	A
**Da Ye Wo Sun**	0.119	A
**Salinas**	0.118	A
**PI 251246**	0.087	AB
**Reine des Glaces**	0.077	AB
**Eruption**	0.039	B

**Roots**	0.098	A
**Stems**	0.089	A

**Early stage of disease progress**	0.122	A
**Late stage of disease progress**	0.095	AB
**Control (healthy, uninoculated plants)**	0.064	B

Additional file S1: Standard curves for *Sclerotia minor* quantification in lettuce tissue.

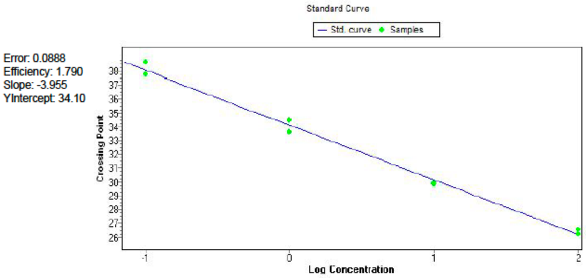

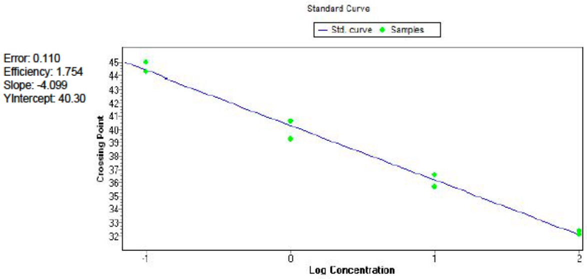

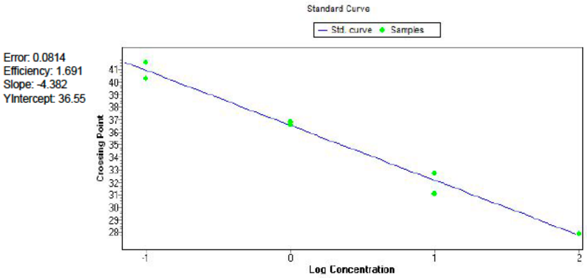

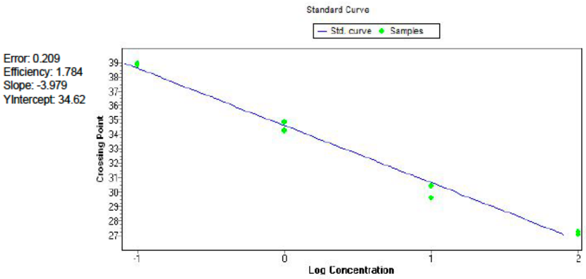

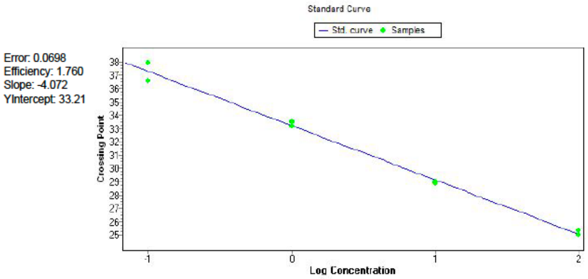

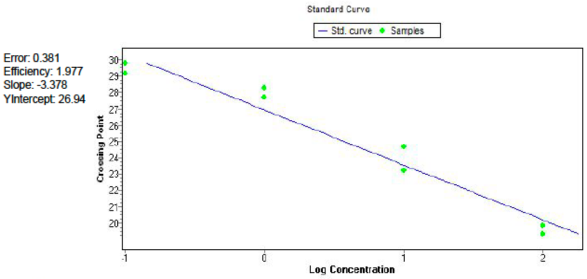

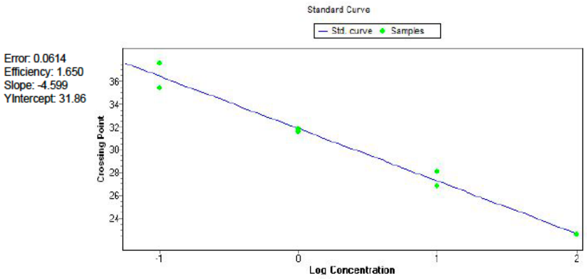

Supplement: Supplementary file 1 — Supplementary Material 1 [file 12870_2024_5399_MOESM1_ESM.pdf]
